# Public health equity in refugee situations

**DOI:** 10.1186/1752-1505-5-6

**Published:** 2011-05-16

**Authors:** Jennifer Leaning, Paul Spiegel, Jeff Crisp

**Affiliations:** 1 FXB Center for Health and Human Rights, Harvard School of Public Health, Boston, MA, USA; 2 Division of Operational Support, United Nations High Commission for Refugees, Geneva, Switzerland; 3 Policy Development and Evaluation Service, United Nations High Commission for Refugees, Geneva, Switzerland

## Abstract

Addressing increasing concerns about public health equity in the context of violent conflict and the consequent forced displacement of populations is complex. Important operational questions now faced by humanitarian agencies can to some extent be clarified by reference to relevant ethical theory. Priorities of service delivery, the allocation choices, and the processes by which they are arrived at are now coming under renewed scrutiny in the light of the estimated two million refugees who fled from Iraq since 2003.

Operational questions that need to be addressed include health as a relative priority, allocations between and within different populations, and transition and exit strategies. Public health equity issues faced by the humanitarian community can be framed as issues of resource allocation and issues of decision-making. The ethical approach to resource allocation in health requires taking adequate steps to reduce suffering and promote wellbeing, with the upper bound being to avoid harming those at the lower end of the welfare continuum. Deliberations in the realm of international justice have not provided a legal or implementation platform for reducing health disparities across the world, although norms and expectations, including within the humanitarian community, may be moving in that direction.

Despite the limitations of applying ethical theory in the fluid, complex and highly political environment of refugee settings, this article explores how this theory could be used in these contexts and provides practical examples. The intent is to encourage professionals in the field, such as aid workers, health care providers, policy makers, and academics, to consider these ethical principles when making decisions.

## Introduction

In the face of global demographic trends and recent political experience, addressing concerns of public health equity in the context of refugee and other forcibly displaced populations has become more complex and challenging. Important operational questions now faced by humanitarian agencies can to some extent be clarified by reference to relevant ethical theory. In conducting such an analysis, this paper seeks to provide a normative as well as practical context for more formal policy deliberation on strategies to address the changing demands on refugee health services worldwide. Much of the debate is relevant to other populations affected by violent conflict including internally displaced persons (IDPs).

For decades, the majority of refugees who required humanitarian protection and services were from poor areas of the developing world in Asia, Africa, and Latin America. When crises occurred, people would flee across international boundaries into equally poor adjacent host countries. The emergency health service needs of these populations, although enormous in the aggregate, were relatively lean when assessed on a per capita basis. The needs of the host populations were similarly constrained by their baseline meagre living conditions and very low economic indicators. In general, it was assumed that everyone--refugees and host populations--were accustomed to subsistence levels of existence, in terms of required inputs for food, water, shelter and basic health care.

In this traditional model of service delivery, the infusion of resources occasioned by the establishment of refugee sites within another country required a measured and delicate strategy towards the local host population. Attention to meeting the needs of local people was considered important even early in the emergency phase, with the dual aim of providing a minimum level of protection and support to the refugees while ensuring some level of equivalence in living conditions and services between the two populations.

These priorities, the allocation choices, and the processes by which they are arrived at, are now coming under renewed scrutiny in the light of the estimated two million refugees who fled from Iraq into host countries elsewhere in the Middle East since 2003. A high proportion of these Iraqi refugees are middle class and their demographic and epidemiological disease profiles reflect the age distribution and burden of chronic disease associated with populations from the developed world. After years of experience in supporting this group of refugees, the humanitarian community is confronting issues of budgetary constraints. These constraints have accelerated the discussion of over-arching issues of service equivalence between host and refugee populations and relative equity (in terms of per capita costs), not just in the context of the Middle East but across the international span of humanitarian refugee operations.

## Operational questions of public health equity in humanitarian situations

Those in the humanitarian community who care for refugees now confront three urgent issues requiring strategic guidance and operational support: 1) What relative priority to give to health among other service responsibilities?; 2) How to allocate resources for health between and within different refugee populations?; and 3) How to identify and justify transition or exit modalities?

### 1) Health as a relative priority

It could be logically argued that much of the operational and ethical concern about allocation decisions could be allayed by a shift in priorities within humanitarian agencies. Were health granted a larger share of humanitarian organisations' budgets, there would be less pressure on making fine-grained choices about who gets what. Many health providers believe that such a shift is necessary and there is increased demand from donors to address refugee health needs, at least for certain populations. However, it is also necessary to come to a consensus on the relative contribution of health to overall individual and population well being compared with the impact of education, livelihoods, and intensified protection efforts.

Concerns about health as a relative priority also prompt closer examination of the extent to which the health care that is delivered meets minimum standards of health services. There may well be considerable room for improvement in provider skills and medical understanding, adherence to standard protocols and interventions, prevention measures, maintenance of adequate supplies of basic medications and materials, procedures for sustained monitoring and follow-up, coordination and referral mechanisms, and management and information systems. This attention to quality would likely require further resources for health, thus driving demand for health care to assume a greater share of the overall humanitarian budget.

### 2) Allocations between and within different refugee populations

Distinctions between refugee and host populations and within refugee populations themselves, in terms of demographic characteristics, income, vulnerabilities and health status, have operational and ethical implications for refugee health policies and programmes, as do differences in the health care delivery policies and capabilities of host states.

#### a) Blurring distinctions between camp, host populations and urban refugees

Humanitarian policies in all sectors, not just health, were generally created to address the needs and concerns of populations in defined locations, often far from urban centres. Two factors have combined to bring about a collapse in whatever urban-rural camp divide might have at one time existed: wars are increasingly encroaching on urban areas where trapped populations, if they move at all, do so within a very circumscribed ambit of densely populated areas and, as the duration of a refugee settlement in a particular area has extended, the host population has increasingly congregated towards the nidus of international activity. In many areas, the geographic, social, and economic boundaries between camp and host settlement have become blurred. This phenomenon has been seen in many situations including refugee settlements in Thailand, Uganda, Zambia, and Yemen.

This growing phenomenon of urban refugees has a myriad of implications for policy and programme. From the health sector perspective, given current humanitarian information systems, personnel, and operational capacity, it is much more difficult for field staff to keep track of people when they move to urban areas, to assure that they are receiving minimum levels of care, to coordinate referrals according to protocol, and to manage the costs attached to whatever services they receive or seek on their own. Furthermore, many refugees are not officially allowed to be living in urban centres. Thus, they remain anonymous and at risk. Additionally, secondary and tertiary care services are more developed in urban settings. Therefore, more complicated and expensive cases often present in urban refugee situations. Typically, for example, chronic diseases are more frequently diagnosed and treated among refugees who have located in urban areas compared with the same group of refugees who have fled a country and are situated in more remote areas.

#### b) Distinctions between refugee and host populations

Sphere standards state that interventions should be designed to close the gap between existing living standards and the Sphere minimum standards [1]. UNHCR's guiding principles for public health state that services provided to refugees should be similar to those provided in the country of origin and host country. However, minimum essential services should be met in all situations [2].

This policy has pragmatic and ethical justification, in that it maintains a sense of fairness and equity between two contiguous groups of people who must, for a range of security and political reasons, be encouraged to live in this adjacency as harmoniously as possible for an indefinite period of time.

Four recent factors, tightly related, accentuate the need to amplify and clarify existing policy relating to this distinction between host and refugee populations. The first is that an increasingly large percentage of refugees are forced to continue to have a refugee status for years, if not decades, and so their health needs are becoming more complex and diverse than can be accommodated by the basic primary health care systems provided by humanitarian agencies and or by those available in the surrounding local host areas. The second factor is that as agencies have become more successful in providing the basic health care package, populations have survived to robust adulthood and enjoy greater life expectancy. This demographic shift is followed by an epidemiological shift that culminates in a third factor; longer life expectancy also moves populations into the age groups where chronic illnesses become more predominant. The fourth factor is the shifting political demographics of refugee flows, whereby refugees from more developed countries with health needs of older populations sometimes seek safety in less developed areas with comparatively inadequate health services. This shift has occurred most recently in the Cote d'Ivoire crisis where refugees from that country have fled into remote areas in Liberia where provision of basic services to the local populations has been a long-standing challenge. A recent article by Larry Gostin urges an international framework for national health systems to meet minimum population survival needs [3]. Were this idea to be taken forward, it would need to account for the eventuality of incoming refugee flows, some of which might well contain populations with more complex needs than the host populations.

#### c) Distinctions across refugee populations

The Iraqi refugee crisis has cast in sharp relief the familiar but now acute dilemma of relative resource allocation across refugee populations. On a per capita basis, the budget for an Iraqi refugee is many times higher per capita cost of providing for a refugee in most parts of Africa or Asia. The concern about relative equity arose earlier with the 1999 Kosovo crisis, when per capita expenditures for Kosovar refugees in Albania and Macedonia, a population that was relatively elderly and burdened with chronic disease, were determined to be many times greater than annual per capita costs for refugees elsewhere in the world [4].

Now that the question is framed in the context of dealing for years with approximately two million Iraqi refugees whose health care needs are costly and demanding, the matter is once again a matter of active interest and debate. Under what circumstances is it acceptable to tolerate large differences in resource allocation between one refugee population, say in Chad, and another, say in Jordan?

#### d) Individual cases as exceptions from population-based protocols

Among many humanitarian providers the allocation decisions that elicit the most intense ethical difficulty are those that address individual cases of extreme and urgent need. The dilemma has until recently most acutely been felt in the context of refugee populations supported by relatively low budgets for primary health care, in poor areas of Africa and some parts of Asia. Respect for standard population-based protocols of care and awareness of grave budgetary constraints collide with the knowledge that expenditure of scarce funds would very likely save the life of an acutely ill or injured child or young adult. Many humanitarian providers, particularly those working with older populations from middle income countries, are also confronted with decisions of approving advanced interventions (e.g. complex surgery, cancer therapy, renal dialysis, thalassemia treatments) that would sustain or salvage the life of a chronically ill and often aged adult [5]. Thus, guidelines for clearly defined standard operating procedures for referral care in such circumstances have been developed [6].

Emergency triage principles in mass casualty events are usually well understood; one must strive to maximise the health of the greatest number of people for whom one is responsible. But in settled refugee contexts this principle would suggest that exceptions requiring expenditures outside of approved budget and protocols of care would have to be carefully defended on non-arbitrary/objective criteria. However, operational ambiguities (e.g. not knowing what has already been expended for health care, what excess the budget might permit, what process to follow for higher level permission, will future funds be available for expensive chronic cases) make a difficult ethical decision even more difficult.

### 3) Transition and exit strategies

The length of time that refugees remain in refugee status now far exceeds the expectations of those who framed and affirmed the Refugee Convention or its Protocol [7]. For example, the average estimated length of stay in a country of asylum has increased from 9 years in 1993 to 17 years in 2003. In most instances, refugees stay in host countries well past the emergency phase of the initial crisis that prompted their forced displacement because a number of political, social, and/or economic barriers prevent their return, local integration or their resettlement.

This long duration of stay has forced the humanitarian community to determine what further elements in a more comprehensive health package it now must assume to provide care for refugee populations who have survived to experience the morbidity patterns of older age. To what extent must humanitarian agencies begin to work with Ministries of Health at the national level to build up secondary and tertiary institutions of care? What are the limits of humanitarian responsibility for health and how might responsibilities of other actors, particularly development actors, be envisioned and promoted?

The need to design a strategy for an effective and sustainable handover from humanitarian agencies to development organisations and Governments is not just a matter for long-stay refugees. Due to global demographic trends, health-relevant distinctions between 'first world' refugee populations and those from the developing world are beginning to erode. Humanitarian agencies need to recognise the ways in which the aging demographics of their entire populations are, from the beginning of their stay, driving the demand for more advanced and sophisticated health care services.

## Ethical guidance for addressing operational questions of public health equity

### 1) Relevant ethical frameworks

A review of the literature suggests that the most relevant normative principles lie in distributional ethics, notions of justice, and decision-making on ethical questions. A number of moral philosophers and social analysts, principal among them John Rawls [8], Norman Daniels [9], and Amartya Sen [10-12] have made major contributions to this literature.

Much of the humanitarian discussion of public health equity relating to refugees focuses on resource allocation, which is a central concern in distributional ethics and notions of justice. Health can be seen as one among many social goods that require resources. Most theorists on social inequalities propose solutions based on the assumption that the pool of resources is finite and that the questions to resolve are how to make re-distributions within that fixed pool. The discussion is thus about how to accomplish transfers of resources from those with access to abundant goods (health) to those without.

Here is where questions of justice or fairness are relevant. The contract theorists, such as Rawls and Daniels, argue that a society must collectively come to some internal agreement about what is a fair and just solution to resource allocation and resource transfers. The capabilities theorists, such as Sen, hold that an essential attribute of a just and fair society is that it makes it possible for each of its citizens to achieve his or her full capabilities. Fortunately, both contract and capabilities theorists can get very practical. They all agree that it is not wise or feasible to try to make everyone in a society equal, in terms of wealth and access to social goods. They reason that resource transfers to achieve absolute equality would abuse the rights of those who are wealthy, would gravely deflate incentive systems, and might introduce new problems (for instance, how would one assure that the redistribution of wealth was spent on important social goods, or would not impair the further production of social goods, or would not get lost if levelled over a vast number of very poor?).

They also both agree that it is not wise or humane to make these resource transfers solely on the basis of marginal utility that is greatest good for the greatest number. The reasoning here is that crude economic cost-benefit analysis (conducted at a population level) overlooks the key ethical question of relative need. The relative value of a resource transfer is not just what it accomplishes at the population level but also what it means to individuals who receive the resource transfer. Thus transfers of resources to the very ill (such as government support for those on dialysis) might not perceptibly raise aggregate measures of population health but would mean a great deal to those individuals and their families who are suffering and to the rest of the population who might anticipate needing those resources were they ever to fall into similar circumstances [13].

Grounds for making ethical decisions are contested and mark a divide among moral philosophers. Is there one unifying rule (as Kant and Rawls would have it) or must people deliberate on the basis of the situation and the evidence, using principles as appropriate (the stance taken by William James [14], Charles Taylor [15], Albert Jonson, Stephen Toulmin [16], and others)? Of practical relevance here is that whichever position one adopts, there will still be the need to agree on a set of deliberative principles, a process framework for arriving at decisions and for achieving support from the large numbers of people who will be affected.

The work of Daniels is particularly important in defining what this process might look like in the context of making decisions about health care allocations. Four major conditions define a fair process for decision making in health allocation:[17]

1. Publicity condition (relating to transparency and accessibility)

2. Relevance condition (evidence-based, assessed as fair by a wide group of stakeholders)

3. Revision and appeals condition (mechanisms for appeals and revisions)

4. Regulative condition (voluntary or public oversight and regulation of the processes)

These conditions do not set forth the content of the decisions (reached through invocation of a unifying rule or through casuistic argument) but in Daniels' view will provide the legitimizing framework for making them. Yet, as he is aware, this process-based approach has been developed from within the framework of one nation-state, with possible application in other states that have similar socio-economic hierarchies and political cultures. Humanitarian agencies must work across and within highly diverse societies, some with good governance but many without.

### 2) Ethical approaches to major operational questions of public health equity in humanitarian situations

#### a) Health as a relative priority

The contract theorists argue that health is important to individuals and to society but that in the context of a liberal and democratic state it is equally if not more important to devote resources to the maintenance of political and civil structures and to the workings of a competitive economic system. The capabilities approach would offer a more profound role for health, arguing that it is a crucial component in allowing an individual to achieve his or her full capabilities, expressed as a sense of agency and wellbeing. Contract theorists would see the state as compensating for inequalities by providing minimum resources to the poor, say for primary health care or for acute catastrophic care; Sen would require the state to provide resources such as adequate food, shelter, water, sanitation, education, as well as more narrowly defined health care inputs, so that the poor were granted the means to become healthy in the first place.

Consequently, for the humanitarian community, the more exploration that is given to the role that health plays in promoting other aspects of the good society, the more health assumes greater priority in the set of primary goods or in the hierarchy of human capabilities. For instance, enhanced investments in secondary obstetrics units and qualified midwives would markedly improve the prospects and wellbeing of entire families who now lose their mother in childbirth. Similarly, providing dental care to elderly might well improve their nutrition and prolong their contribution to society. In some refugee programmes, multi-sectoral integrated activities [18] are promoted to partially address this issue.

#### b) Resource allocations within and across refugee populations

Within one refugee population, the argument from justice and fairness would suggest that emphasis be placed on raising the health status of those most in need, but the extra resources required to do so for this one group within one refugee population could not be extracted if doing so imposed a significant loss to those who were receiving less per capita.

The consensus from both the contractual and capabilities approach is that within-system differences are tolerable to the extent that those at the bottom receive an appropriate minimum bundle of services that provide essential primary goods or human capabilities. Both approaches would hold that, as with any social good, including health, such a minimum might vary from one society to another.

For populations of refugees from different countries, there is no ethical requirement that humanitarian agencies take an egalitarian approach. It is fair and just to establish social minimums and the content and expense of those social minimums may vary depending upon need and the level of primary goods and capabilities to which that population is accustomed. The upper limit on those resources would be reached when within a fixed budget the transfer of funds begins to impinge on the wellbeing of those who are basically healthy or who are accustomed to managing at a lower social minimum.

Another approach would be to frame the question as one of international health disparities - to what extent are these unjust and to what extent can a health-based approach resolve these injustices? From one perspective, the regime of international justice has not developed to the extent that one can identify international obligations to address effectively these cross-state disparities at the international level. Yet a more amplified reading of international justice obligations raises a real practical as well as ethical dilemma for humanitarian agencies. Given widespread adoption by nation-states of international human rights and humanitarian law, the establishment of UN humanitarian agencies, and a panoply of expressed international commitments and contributions to alleviating world poverty and misery, one could in fact infer that humanitarian agencies might have some responsibility for addressing and redressing these disparities, to the extent they are socially controllable (and many health disparities are very much so). Yet even UNHCR, an international institution with a legal mandate to care for all refugees in the world, plays in that intermediate zone where, according to political philosophers, it has neither the machinery of the state nor the legitimacy of political power to define the hard choices or to undertake their resolution.

To make room for exceptions from resource allocation protocols, which in ethical terms is always an absolutely valid and important demand on a population-based health care system aligned according to principles of population ethics, several process and system supports would need to be in place. But the basic ethical finding from the literature is that the obligation to deal with exceptions, with individual cases, does not go away when one moves from individual care based on medical ethics to population care based population ethics. In fact, the medical ethicist would assert that the moment you hear about this case, you must act at least in a dual role, as a clinician whose primary responsibility is beneficence and as a manager in a rationed system.

In the process path of making exceptions, the advice and guidance from stakeholders, including members from different refugee and host communities and possibly donors, would be most valuable in framing and legitimating options. Fairness issues would demand the highest level of transparency, so that everyone involved at all phases would know what was possible to permit as an exception and what was not.

### 3) Examples of applying ethical approaches to public health refugee situations

Given UNHCR's recent experience in addressing the needs of Iraqi refugees [19] combined with the agency's push to tackle the complex issues of urban refugees [20], practical operational guidance using lessons learned has been developed that has attempted to use some of the ethical principles discussed above. *Access *to quality health care services in all refugee settings in similar ways and at similar or lower costs to that of nationals has become a major principle combined with *equity *(i.e. establish special assistance arrangements for vulnerable refugees and individuals with specific needs so that they can access services equitably) and *prioritisation *(i.e. ensure refugees access to essential primary health care services and emergency care, and ensure that these take precedence over referral to more specialised medical care). Avoidance of parallel systems that provide different services to refugees than to existing services for national populations is stressed. Rather, the new guidance urges UNHCR and its partners to advocate that public health services for refugees and asylum seekers are made sustainable by being *integrated *within the national public system whenever feasible. UNHCR may draw on partners to temporarily provide services complementary to government services where there are significant gaps in service provision or when services are of insufficient quality.

For example, UNHCR has recently negotiated with the Government of Iran to undertake a health insurance scheme that would provide over one million refugees with a level of access to secondary and tertiary care that is similar to that of an "average" Iranian. In Iran, registered Afghan refugees have access to primary health care services in the same manner as that of Iranians. Furthermore, they have the right to work and most families have access to some sort of income. The health insurance scheme is voluntary and relies on the Afghan refugees to pay a monthly premium and co-payment of 30% of any hospitalisation. In order to address those Afghan refugees who cannot afford the premiums and co-payments, UNHCR is working with the Government of Iran to develop criteria as to who would be considered vulnerable and then pay for their premiums and part of the co-payments. Such a large scale insurance scheme for refugees has never been undertaken before and its implementation and results will have major implications for other countries where refugees have sufficient income to pay for such services.

During the Iraqi refugee crisis, chronic diseases and expensive tertiary care became a major issue. UNHCR developed an Exceptional Care Committee that assesses individual cases and makes objective decisions about the referral based primarily on prognosis and cost. This committee is professional and independent in its decision making. The committee is equipped with guidance on review criteria. The composition of the referral committee depends upon the country setting. Based on experience and wherever feasible, it is recommended that the referral committee include a minimum of three health professionals to ensure a fair and transparent process that addresses both the reality of health services in the country and the best evidence-based practices. It is also recommended that the medical technical decisions for referral, which are based primarily on prognosis, take place sequentially, before decisions are made based on financial considerations [21]. Many other countries that were dealing with refugee referral but did not have standard operating procedures have now implemented some sort of guidelines to ensure objectivity and transparency. Using refugees with a medical background as communicators and facilitators has greatly improved understanding and compliance with referral processes. However, the rejection of referral for persons who have a disease with a very poor prognosis to give priority to another with a better prognosis remains a traumatic experience for the refugee, his/her family, and the engaged staff at UNHCR and its partners.

## Conclusion

This proposed ethical guidance, based on an eclectic selection from overlapping systems of thought and argument, finds that the public health equity issues faced by the humanitarian community can be framed as issues of resource allocation and issues of decision-making. The ethical approach to resource allocation in health requires taking adequate steps to reduce suffering and promote wellbeing, with the upper bound being to avoid harming those at the lower end of the welfare continuum. Exceptions to protocols are allowed and must be taken seriously, according to transparent and informed processes. User fees are not in themselves unethical but difficult to implement ethically in emergency situations. Deliberations in the realm of international justice have not provided a legal or implementation platform for reducing health disparities across the world, although norms and expectations, including within the humanitarian community, may be moving in that direction.

## Competing interests

JL, none; JC and PS are both employed by UNHCR but report no competing interests with regard to this article.

## Authors' contributions

JL did the research and wrote the first draft; PS and JC helped refine the main questions, supplied references and documents as needed, reviewed and helped rewrite subsequent drafts. All authors read and approved the final manuscript.

## Funding

JL prepared a report to UNHCR on issues of public health equity of particular relevance to that agency and received funding for that report. No funding was received by the authors for the preparation of this paper, which is partially based on research undertaken for the longer UNHCR report.

## Ethics review

No ethics committee review of this article is required.
